# Prognostic and immune-related value of *STK17B* in skin cutaneous melanoma

**DOI:** 10.1371/journal.pone.0263311

**Published:** 2022-02-16

**Authors:** Xueying Shi, Qi Zhou, Bingqian Huang, Shilin Xia, Yuankuan Jiang, Shifeng Fang, Jingrong Lin

**Affiliations:** 1 Clinical Laboratory of Integrative Medicine, The First Affiliated Hospital of Dalian Medical University, Dalian, Liaoning, China; 2 Institute (College) of Integrative Medicine, Dalian Medical University, Dalian, Liaoning, China; 3 Department of Dermatology, The First Affiliated Hospital of Dalian Medical University, Dalian, Liaoning, China; 4 Department of Ophthalmology, The First Affiliated Hospital of Dalian Medical University, Dalian, Liaoning, China; Duke University School of Medicine, UNITED STATES

## Abstract

Skin cutaneous melanoma (SKCM) is a common cancer of which mortality is increasing continuously. Our study conducted a series of analyses on the clinical significance of Serine/threonine kinase 17B (STK17B) in SKCM to provide a new biomarker for diagnosis and treatment. The RNA-sequence data were obtained from The Cancer Genome Atlas and Genotype-Tissue Expression databases. The data of 468 SKCM patients were divided into STK17B high- and low-expression groups and analyzed by Bioconductor package to identify the differential expressed genes. The R package of “clusterProfiler” was used for Gene Ontology, Kyoto Encyclopedia of Genes and Genomes, and Gene-Set Enrichment Analysis analyses. A protein-protein interaction network and immune infiltration landscape were respectively constructed via STRING database and ssGSEA. STK17B had lower expression in SKCM than normal tissues. Besides, STK17B expression was significantly related to some clinicopathological characteristics in SKCM patients including T stage, Breslow depth, radiation therapy, melanoma Clark level, and pathologic stage. The Kaplan-Meier curve analyses revealed that the low expression of STK17B was correlated with poor overall survival and disease-specific survival. We constructed nomograms to predict the 1-, 3-, and 5-year survival of SKCM patients. The function enrichment analyses showed STK17B-related differential expressed genes were enriched in cellular differentiation and immune-related progress. STK17B expression level were positively correlated with infiltrating level of immune cells. In this study, we found that STK17B, which played an important role in immune infiltration, could be a new biomarker for diagnosis and prognosis in SKCM patients.

## 1. Introduction

Skin cutaneous melanoma (SKCM) is one of the most malignant skin tumors, which can both locally invade surrounding tissues as well as metastasize systemically. The global incidence of SKCM continues to increase and it is particularly common in fair-skinned populations [1]. The morbidity of SKCM is only 4% to 11% of all skin cancers but the mortality of SKCM is close to three quarters of total fatality rate from skin tumors [2]. As is known, a main risk factor of melanoma is ultraviolet radiation. However, SKCM can also appear in non-sun-exposed area, such as feet, mouth, and nasal passages, caused by dysplastic naevi or hereditary factors [3]. SKCM can be cured through surgical excision if diagnosed in early stage. Immunotherapy and targeted therapies are also used clinically. However, untreated stage IV patients survive less than one year, it is urgent to find novel and effective targets for clinical research and therapeutic method [4].

Serine/threonine kinase 17B (STK17B), also known as DAP kinase-related apoptosis-inducing protein kinase 2 (DRAK2), is located on chromosome 2 (2q32.3). As a member of DAPK family, that all members have been reported inducing apoptosis through abnormal expression in various cell types, STK17B is still in dispute for its effect on apoptosis. STK17B was also a negative regulator of TGF-β signaling, which is known as a crucial step in the tumorigenic development, inhibiting the phosphorylation of R-Smads through its interaction with TβRI. Additionally, STK17B was over-expressed in basal-like and HER2-enriched breast cancer, and silence of STK17B retarded tumorigenesis and tumor growth in xenograft model [5]. STK17B is upregulated in hepatocellular carcinoma tissues and cell lines. At the same time, this gene has been identified facilitating carcinogenesis and metastasis [6]. Besides, there is few literature about the relationship of STK17B and melanoma.

In this study, we indicated the clinical diagnosis and prognostic value of STK17B in SKCM. Firstly, we downloaded the RNA-Seq data from The Cancer Genome Atlas (TCGA) and Genotype-Tissue Expression (GTEx) database, and explored a correlation between STK17B and SKCM. A contrastive analysis of STK17B differential expression was performed between SKCM and normal tissues. The diagnostic and prognostic value of this gene were estimated and nomograms were drawn to predict the 1-, 3-, 5-year overall survival (OS), disease-specific survival (DSS), and progression-free interval (PFI) of SKCM patients. The immune infiltration landscape of STK17B was quantified by single-sample gene set enrichment analysis (ssGSEA) method. Furthermore, the SKCM patient samples were divided into high- and low-expression groups according to the expression level of STK17B. The STK17B-related differential expressed genes (DEGs) were identified by comparing the sequencing data of the two groups. Subsequently, the Gene Ontology (GO) and Kyoto Encyclopedia of Genes and Genomes (KEGG) enrichment analyses was performed for STK17B-related DEGs by R package. Gene set enrichment analysis (GSEA) to filtrate significantly enriched gene sets. In addition, we constructed a protein-protein interaction (PPI) network of STK17B. The present study showed that lower STK17B expression was related to poor prognosis and can be considered as a potential diagnostic and prognostic biomarker of SKCM.

## 2. Materials and methods

### 2.1. RNA-sequencing patient data and expression analysis

The RNA-Seq data of TCGA and GTEx database were downloaded from the University of California Santa Cruz (UCSC) XENA (https://xenabrowser.net/datapages/), which were handled by the Toil process and transformed into transcripts per million reads [7]. The expression of STK17B in various types of cancers was analyzed. Patients with unavailable clinical information were excluded. In addition, total of 1282 normal and SKCM samples with detailed clinicopathological information were collected and further analyzed.

### 2.2. A receiver operating characteristic (ROC) analysis and survival analysis

A receiver operating characteristic (ROC) curve analysis was performed to evaluate the diagnostic effectivity for SKCM, and the area under the curve was calculated to assess value of the predictive value of the testing method. According to the median value of STK17B mRNA expression, patients were divided into STK17B high- and low-expression groups. Subsequently, the R package of “survminer” was used to assess the prognostic value of STK17B for the OS and DSS in SKCM. Furthermore, the prognostic value of STK17B was analyzed in subgroups of SKCM patients with different clinicopathologic characteristics, such as pathologic stage, radiation therapy, gender, age.

### 2.3. Construction of nomogram and forest plots

According to the results of Cox regression, nomogram models were constructed to calculate the predictive value of STK17B in estimating the prognosis of SKCM patients. Calibration plots were also performed to assess the effectiveness of the model. In addition, forest plots were constructed to visualize the prognostic value of STK17B for OS, DSS, and PFI in SKCM patients.

### 2.4. Analysis of immune infiltration

The TCGA gene expression dataset was used to quantify immune infiltration landscape of STK17B by ssGSEA method, and the marker genes of 24 immune cells types were previously reported [8]. The ssGSEA analysis was performed by GSVA package from R program (http://www.bioconductor.org) [9]. The correlation between immune cells and STK17B was analyzed by Spearman’s rank-correlation coefficient [10]. Infiltration levels of immune cells between STK17B high- and low-expression group was compared by Wilcoxon rank sum test [11]. The R package estimate was used to analyze the relationship of immune score and STK17B expression [12]. Six immune subtypes were identified according to different immune expression signatures of tumor, including C1 (wound healing), C2 (IFN-γ dominant), C3 (inflammatory), C4 (lymphocyte depleted), C5 (immunologically quiet), and C6 (TGF-β dominant) [13]. We also analyzed STK17B expression in different immune subtypes using TISIDB (http://cis.hku.hk/TISIDB/index.php) [14, 15].

### 2.5. Analysis of STK17B-related DEGs for SKCM between STK17B high- and low-expression groups

The Bioconductor package “DESeq2” was used to compare the RNA-seq of STK17B high- and low-expression groups and identify STK17B-related DEGs in SKCM [16]. The adjusted *P* < 0.05 and |log_2_ Fold change (FC)| >2 were selected as cut-off criteria to identify STK17B-related DEGs. A volcano plot and heat map were drawn using ggplot2 packages of R for the visualization of the identified STK17B-related DEGs.

### 2.6. Functional enrichment analysis

The clusterProfiler package was used to perform GO and KEGG analysis of STK17B-related DEGs [17]. GO is a comprehensive source of digital data relating to the functions of genes in three independent categories: molecular function, biological process, and cellular component. The significant P-value was adjusted by Benjamin and Hochberg method.

### 2.7. Gene set enrichment analysis (GSEA) and protein-protein interaction (PPI) network

GSEA is a computational method to identify significantly enriched or depleted groups of genes. GSEA was performed by R package of clusterProfiler based on the STK17B-related DEGs [17]. In this study, GSEA was used to identify significantly enriched gene sets between STK17B high- and low-expression groups. The gene sets with a nominal *P* < 0.05 and a false discovery rate < 0.05 were considered as significantly enrichment. We analyzed the PPI network of STK17B via the STRING database (https://string-db.org/), and the minimum required interaction cutoff is 0.4 [18]. Subsequently, the PPI network was constructed by Cytoscape software (3.8.0) [19].

## 3. Results

### 3.1. The expression levels of STK17B

**Fig 1:**
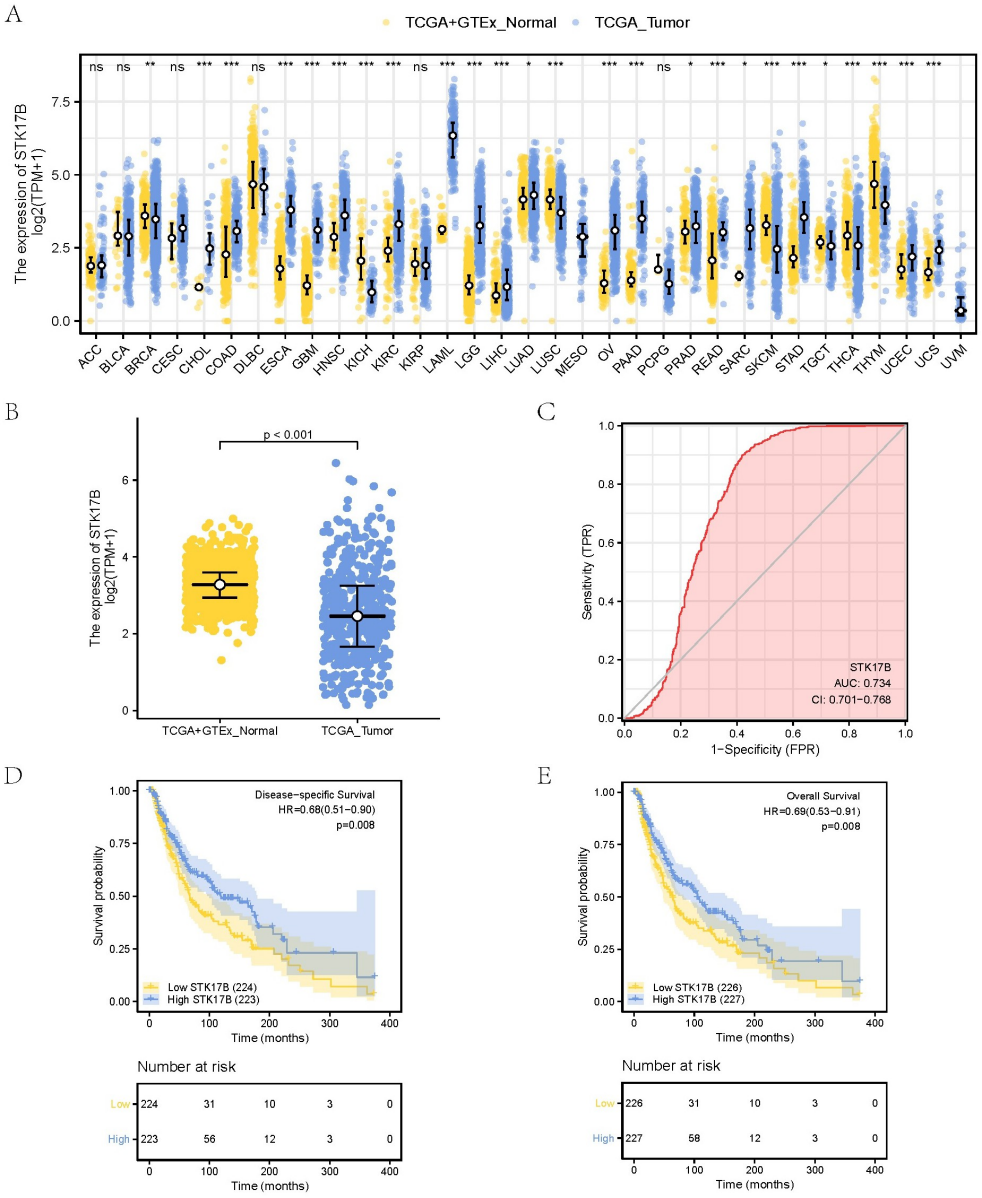
STK17B expression and prognosis in skin cutaneous melanoma (SKCM) patients. (**A**) The association between STK17B expression in various types of cancers; (**B**) STK17B has low expression in SKCM tissues; (**C**) the diagnostic value of STK17B in SKCM patients by receiver operating characteristic curve analysis; (**D, E**) the prognostic value of STK17B for disease-specific survival and overall survival in SKCM patients.

We explored the expression differences of STK17B in 33 human cancers and corresponding normal tissues based on the TCGA and UCSC data sets. The association between STK17B expression in various types of cancers was analyzed (Fig 1A). Compared to normal samples, dramatically lower expression of STK17B was showed in SKCM (Fig 1B). Furthermore, we evaluated the diagnostic value of STK17B in SKCM patients by ROC curve analysis (Fig 1C). the area under the curve value of the ROC curve of STK17B was 0.734 (95% confidence interval [CI] = 0.701–0.768), suggesting that normal tissues could be effectively distinguished from SKCM tissues according to the expression level of STK17B.

### 3.2. Correlation between STK17B expression and clinicopathological characteristics in SKCM

The correlation between the STK17B expression and clinicopathological characteristics of SKCM patients was analyzed (Table 1). The correlation results indicated that STK17B expression was significantly associated with T stage, Breslow depth and Radiation therapy in SKCM patients (*P* = 0.013, *P* = 0.009, *P* <0.001, respectively). However, the results showed that the STK17B expression were not significantly associated with other parameters, including N, M stage, pathologic stage, melanoma Clark level, melanoma ulceration, gender, race, tumor tissue site, BRAF status and age (all *P* > 0.05).

**Table 1:** The correlation between the STK17B expression and clinicopathological characteristics of skin cutaneous melanoma patients.

Characters	level	Low expression of STK17B	High expression of STK17B	P
**n**		234	234	
**T stage (%)**	**T1**	16(8.6%)	25(14.4%)	0.013
	**T2**	39(20.9%)	39(22.4%)	
	**T3**	39(20.9%)	51(29.3%)	
	**T4**	93(49.7%)	59(33.9%)	
**N stage (%)**	**N0**	116(56.0%)	118(57.8%)	0.708
	**N1**	39(18.8%)	35(17.2%)	
	**N2**	22(10.6%)	27(13.2%)	
	**N3**	30(14.5%)	24(11.8%)	
**M stage (%)**	**M0**	210(94.6%)	206(94.5%)	1.000
	**M1**	12(5.4%)	12(5.5%)	
**Pathologic stage (%)**	**Stage I**	32(15.4%)	44(21.9%)	0.197
	**Stage II**	80(38.5%)	60(29.9%)	
	**Stage III**	85(40.9%)	85(42.3%)	
	**Stage IV**	11(5.3%)	12(6.0%)	
**Melanoma Clark level (%)**	**I**	4(2.5%)	2(1.2%)	0.057
	**II**	6(3.8%)	12(7.5%)	
	**III**	29(18.2%)	47(29.4%)	
	**IV**	91(57.2%)	77(48.1%)	
	**V**	29(18.2%)	22(13.8%)	
**Breslow depth (%)**	**< = 3**	80(44.4%)	104(58.8%)	0.009
	**>3**	100(55.6%)	73(41.2%)	
**Melanoma ulceration (%)**	**No**	70(42.4%)	75(51.4%)	0.143
	**Yes**	95(57.6%)	71(48.6%)	
**Radiation therapy (%)**	**No**	206(90.0%)	175(75.4%)	<0.001
	**Yes**	23(10.0%)	57(24.6%)	
**Gender (%)**	**Female**	90(38.5%)	89(38.0%)	1.000
	**Male**	144(61.5%)	145(62.0%)	
**Race (%)**	**Asian**	9(4.0%)	3(1.3%)	0.086
	**Black or African American**	0(0.0%)	1(0.4%)	
	**White**	218(96.0%)	227(98.3%)	
**Tumor tissue site (%)**	**Extremities**	109(51.4%)	86(42.2%)	0.126
	**Head and Neck**	20(9.4%)	17(8.3%)	
	**Other Specify**	4(1.9%)	9(4.4%)	
	**Trunk**	79(37.3%)	92(45.1%)	
**BRAF status (%)**	**Mutant**	106(45.5%)	126(54.3%)	0.071
	**Wild Type**	127(54.5%)	106(45.7%)	
**Age (median [IQR])**		58.00[48.25,71.00]	60.00[46.25,70.00]	0.444

### 3.3. STK17B is an independent predictor of prognosis in SKCM

The effects of STK17B on the DSS and OS of SKCM patients were analyzed using the Kaplan-Meier curve (Fig 1D and 1E). The results showed that lower expression of STK17B was associated with worse OS (HR = 0.69(0.53–0.91), *P* = 0.008) and DSS (HR = 0.68(0.51–0.90), *P* = 0.008). In the univariate Cox regression analysis, T, N stage, pathologic stage, melanoma Clark level, Breslow depth, melanoma ulceration, age, race, STK17B were all associated with OS (Table 2). TNM stage, pathologic stage, melanoma Clark level, Breslow depth, melanoma ulceration, age, STK17B, were all associated with DSS (Table 3). The variables with *P* < 0.1 in the univariate analyses were included in the subsequent multivariate Cox regression analysis. Multivariate analyses demonstrated that N stage, Breslow depth, STK17B expression were independent prognostic factors in OS (*P* < 0.05) (Table 2). Similarly, N stage, Breslow depth, STK17B were independent prognostic factors in DSS (*P* < 0.05) (Table 3). Therefore, low STK17B expression was an independent risk associated with poor prognosis in SKCM patients.

**Table 2:** Univariate and multivariate Cox regression analysis of patients’ overall survival prediction based on STK17B expression.

Characteristics	Total(N)	HR (95% CI) Univariate analysis	P value Univariate analysis	HR (95% CI) Multivariate analysis	P value Multivariate analysis
**T stage (T3&T4 vs. T1&T2)**	358	2.040(1.468–2.836)	<0.001	0.956(0.541–1.688)	0.876
**N stage (N1&N2&N3 vs. N0)**	399	1.711(1.271–2.304)	<0.001	3.519(1.030–12.019)	0.045
**M stage (M1 vs. M0)**	427	1.734(0.915–3.287)	0.092	1.886(0.629–5.656)	0.258
**Pathologic stage (Stage III &Stage IV vs. Stage I &Stage II)**	407	1.579(1.177–2.118)	0.002	0.628(0.179–2.204)	0.468
**Melanoma Clark level (IV&V vs. I&II&III)**	312	2.117(1.472–3.045)	<0.001	1.229(0.746–2.024)	0.419
**Breslow depth (>3 vs. < = 3)**	352	2.593(1.892–3.553)	<0.001	1.757(1.026–3.010)	0.040
**Melanoma ulceration (Yes vs. No)**	310	2.087(1.494–2.916)	<0.001	1.456(0.954–2.220)	0.081
**Radiation therapy (Yes vs. No)**	447	0.953(0.674–1.348)	0.785		
**Age (>60 vs. < = 60)**	453	1.678(1.266–2.225)	<0.001	1.242(0.820–1.879)	0.306
**Gender (Male vs. Female)**	453	1.164(0.872–1.554)	0.301		
**Race (White vs. Asian &Black or African American)**	443	0.223(0.103–0.483)	<0.001	0.474(0.063–3.554)	0.468
**Tumor tissue site (Extremities vs. Trunk)**	354	1.063(0.782–1.443)	0.698		
**BRAF status (Mutant vs. Wild Type)**	450	0.774(0.589–1.017)	0.066	0.785(0.524–1.176)	0.241
**STK17B (High vs. Low)**	453	0.694(0.530–0.910)	0.008	0.636(0.430–0.941)	0.024

**Table 3:** Univariate and multivariate Cox analysis of patients’ disease-specific survival prediction based on STK17B expression.

Characteristics	Total(N)	HR (95% CI) Univariate analysis	P value Univariate analysis	HR (95% CI) Multivariate analysis	P value Multivariate analysis
**T stage (T3&T4 vs. T1&T2)**	353	1.842(1.308–2.594)	<0.001	0.904(0.508–1.608)	0.731
**N stage (N1&N2&N3 vs. N0)**	393	1.620(1.179–2.227)	0.003	4.743(1.079–20.844)	0.039
**M stage (M1 vs. M0)**	421	2.013(1.059–3.828)	0.033	1.943(0.654–5.770)	0.232
**Pathologic stage (Stage III &Stage IV vs. Stage I &Stage II)**	402	1.495(1.093–2.045)	0.012	0.439(0.097–1.980)	0.284
**Melanoma Clark level (IV&V vs. I&II&III)**	307	2.075(1.419–3.035)	<0.001	1.329(0.791–2.233)	0.283
**Breslow depth (>3 vs. < = 3)**	347	2.213(1.580–3.099)	<0.001	1.718(1.001–2.949)	0.050
**Melanoma ulceration (Yes vs. No)**	306	1.949(1.369–2.775)	<0.001	1.435(0.931–2.213)	0.102
**Radiation therapy (Yes vs. No)**	441	0.966(0.667–1.400)	0.856		
**Age (>60 vs. < = 60)**	447	1.728(1.278–2.337)	<0.001	1.205(0.781–1.859)	0.399
**Gender (Male vs. Female)**	447	1.151(0.847–1.564)	0.368		
**Race (White vs. Asian &Black or African American)**	437	0.456(0.144–1.450)	0.183		
**Tumor tissue site (Extremities vs. Trunk)**	350	1.086(0.783–1.505)	0.622		
**BRAF status (Mutant vs. Wild Type)**	444	0.785(0.586–1.051)	0.104		
**STK17B (High vs. Low)**	447	0.678(0.508–0.904)	0.008	0.595(0.396–0.894)	0.013

### 3.4. The relationship of STK17B and prognosis of SKCM patients with different clinicopathological status

**Fig 2:**
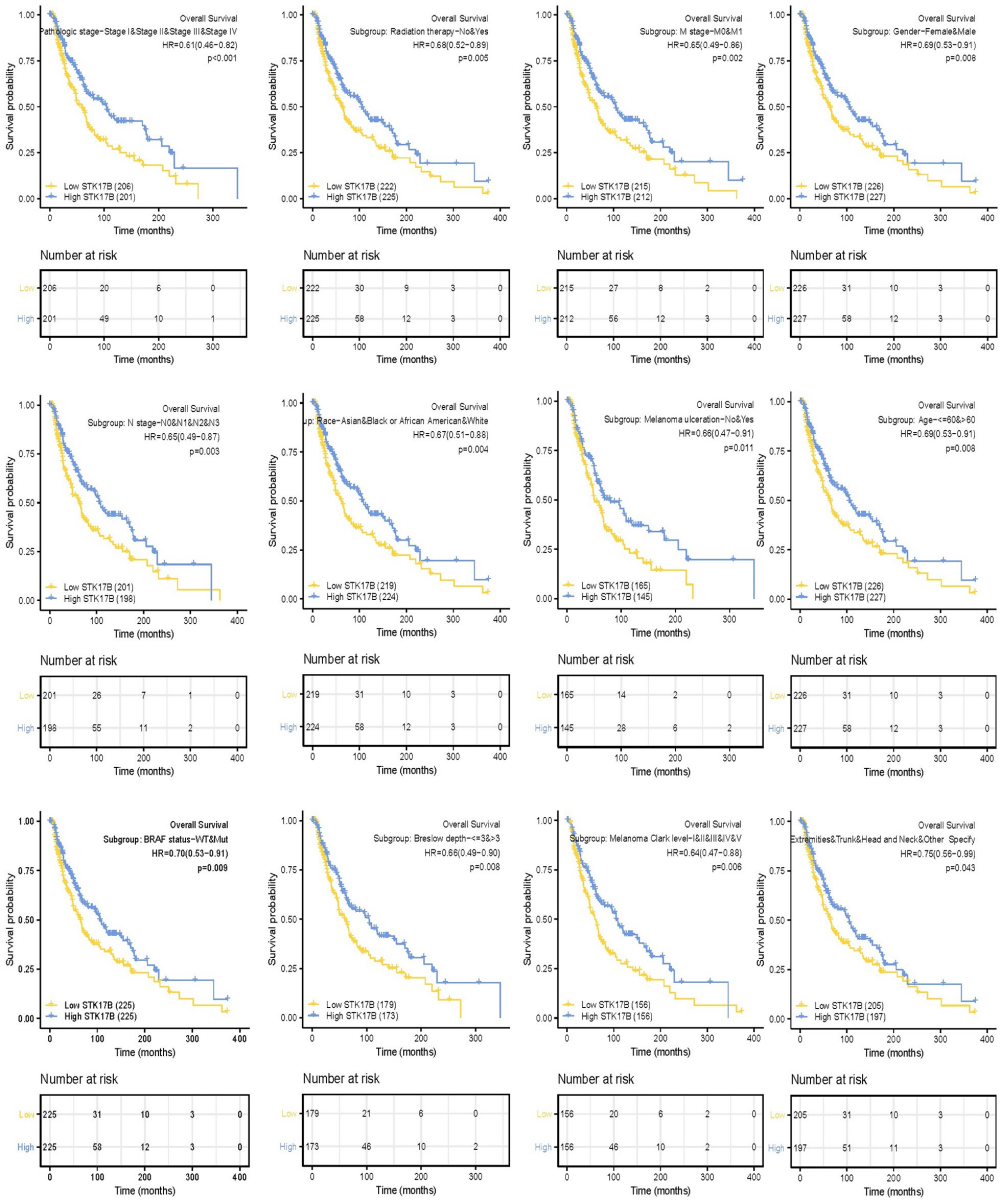
The influence of STK17B and other clinicopathologic characteristics on the overall survival in skin cutaneous melanoma patients.

**Fig 3:**
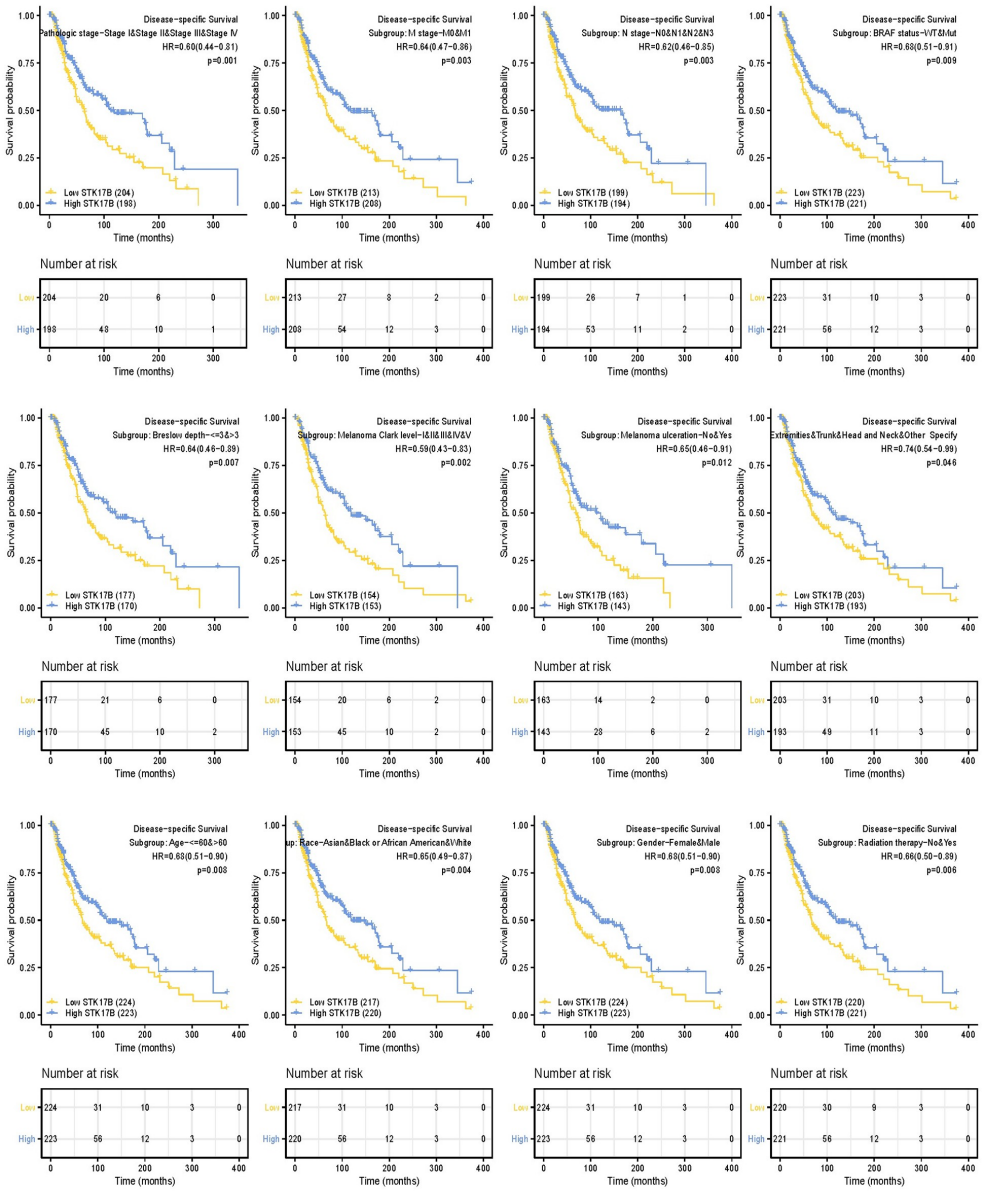
The influence of STK17B and other clinicopathologic characteristics on the disease-specific survival in skin cutaneous melanoma patients.

**Fig 4:**
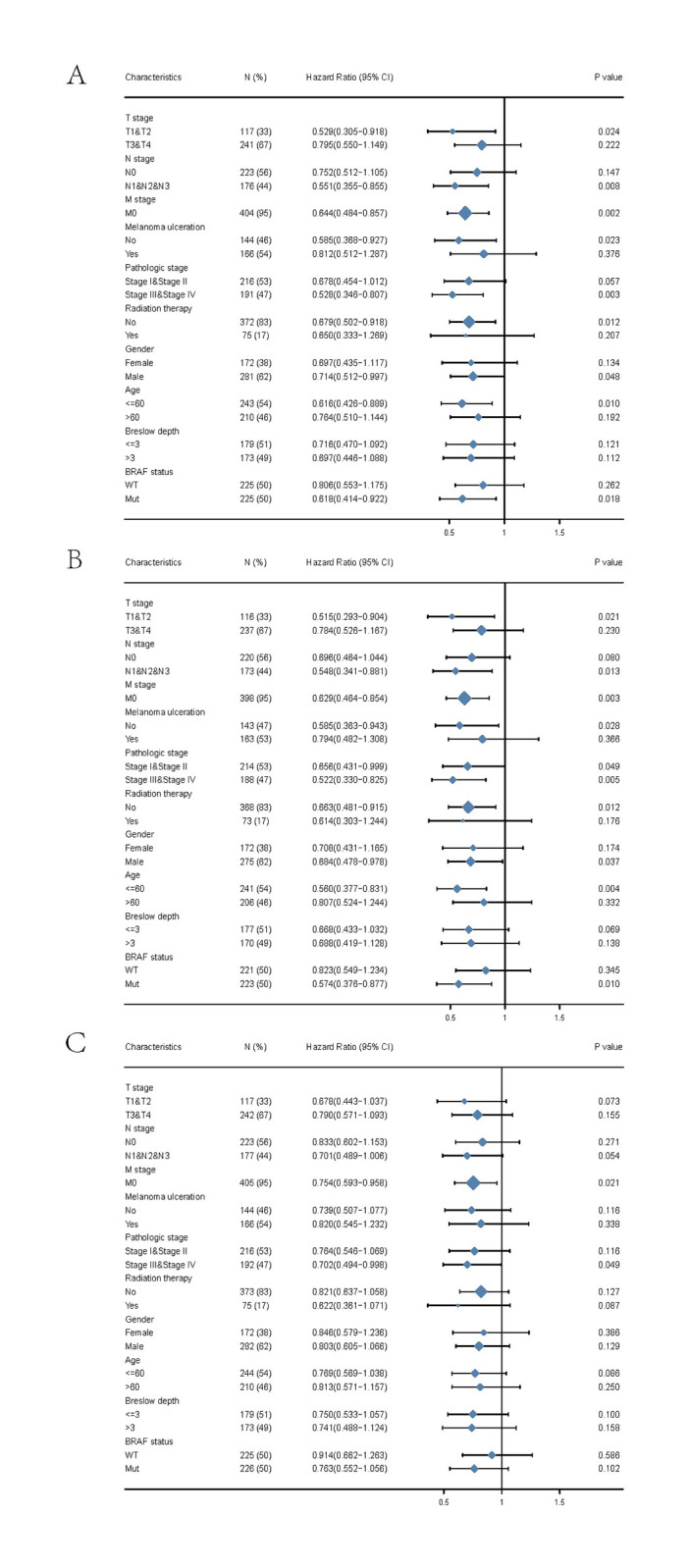
Prognostic value of STK17B in skin cutaneous melanoma subgroups. Multivariate analysis in overall survival (**A**), disease-specific survival (**B**), and progression-free interval (**C**) of SKCM project.

A further subgroup analysis showed that low STK17B was correlated with worse OS in N and M Stage, pathologic stage, radiation therapy, gender, race, melanoma ulceration, age, melanoma Clark level, Breslow depth, tumor site, BRAF status of SKCM patients (Fig 2). Low STK17B was similarly correlated with worse DSS in T and M Stage, pathologic stage, melanoma Clark level, Breslow depth, melanoma ulceration, tumor site, BRAF status, age, race, gender, radiation therapy of SKCM patients (Fig 3). In addition, the prognostic value of STK17B in SKCM subgroups was visualized (Fig 4). There were significant differences in subgroups of TNM stage, melanoma ulceration, pathologic stage, radiation therapy, gender, age, Breslow depth, BRAF status, indicating that STK17B expression level could impact the prognosis in SKCM patient with different pathological stages.

### 3.5. Nomogram construction based on STK17B expression and clinicopathological factors

**Fig 5:**
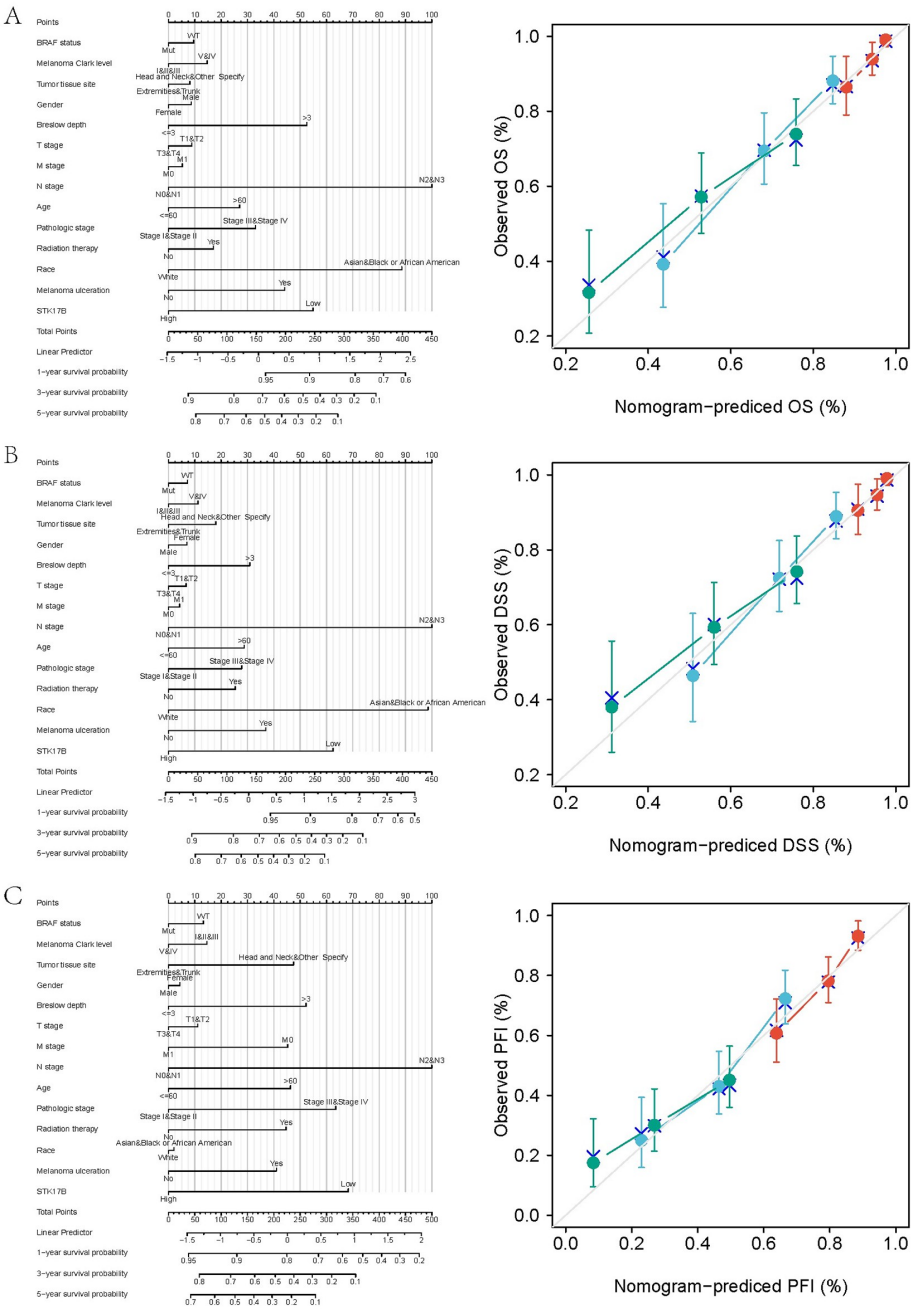
Nomograms of prognostic model and calibration curve. Nomogram models of overall survival (**A**), disease-specific survival (**B**), and progression-free interval (**C**) with respect to the BRAF status, melanoma Clark level, tumor tissue site, gender, Breslow depth, T stage, M stage, N stage, age, pathologic stage, radiation therapy, race, melanoma ulceration and STK17B gene expression.

Based on the results of multivariate analyses with the Cox regression model, we integrated both STK17B expression and other clinicopathological prognostic factors, then constructed nomograms to better predict 1-year, 3-year and 5-year OS, DSS, and PFI of SKCM patients (Fig 5). Higher total points indicated a worse outcome. The C-index for OS, DSS, and PFI prediction were 0.699(0.673–0.725), 0.696(0.668–0.724), and 0.685(0.661–0.708) respectively. The calibration curve for the probability of survival at 1-, 3-, and 5-year showed good agreement between the prediction by nomogram and actual observation. These nomogram-based results demonstrated a good accuracy for predicting the 1-, 3-, or 5-year survival of SKCM patients.

### 3.6. The correlation between STK17B expression and tumor-infiltrating immune Cells

**Fig 6:**
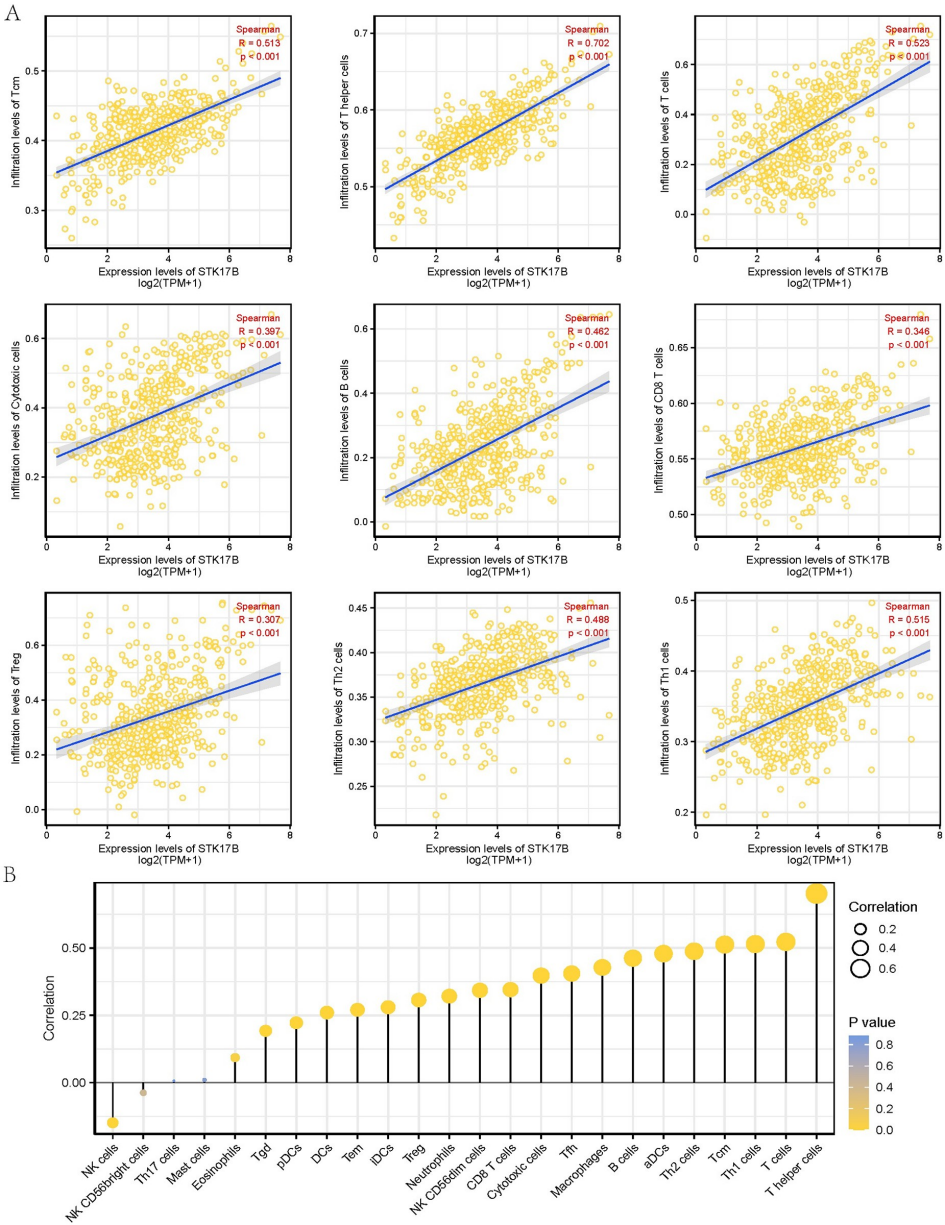
The correlation between infiltration levels and STK17B expression in SKCM patients. (**A**) STK17B expression was positively correlated with the infiltration levels of CD8 T cells, Treg, Th2 cells, Th1 cells, Tcm, T helper cells, T cells, Cytotoxic cells (CD8+), and B cells; (**B**) the relationship of STK17B expression and the infiltration levels of 24 types immune cells.

**Fig 7:**
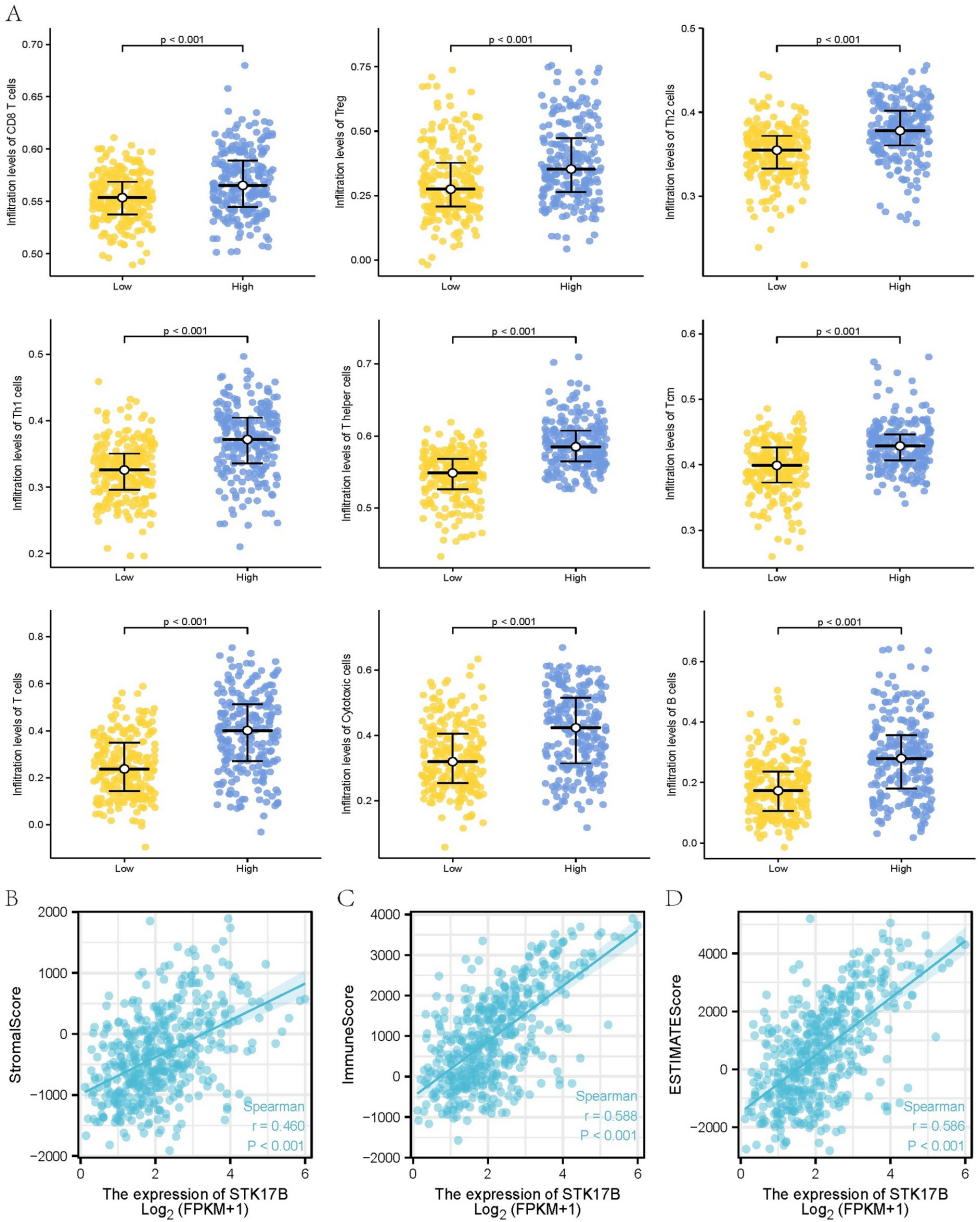
Infiltration level of immune cells and immune score analysis. (**A**) Comparison of different immune cells in STK17B high- and low-expression groups in the SKCM cohorts from The Cancer Genome Atlas database; the correlation between STK17B expression and stromal score (**B**), immune score (**C**) and ESTIMATE score (**D**).

We analyzed 24 types of infiltrating immune cells by ssGSEA method in SKCM, and the association between STK17B expression and 24 types of infiltrating immune cells by Spearman correlation. The results showed that STK17B expression level was significantly positively correlated with infiltrating levels of CD8 T cells (R = 0.346, *P* <0.001), Treg (R = 0.307, *P* <0.001), Th2 cells (R = 0.488, *P* < 0.001), Th1 cells (R = 0.515, *P* < 0.001), Tcm (R = 0.513, *P* < 0.001), T helper cells (R = 0.702, *P* < 0.001), T cells (R = 0.523, *P* < 0.001), Cytotoxic cells (CD8+) (R = 0.523, *P* < 0.001), B cells (R = 0.462, *P* < 0.001) (Fig 6A). It has been revealed the relationship of STK17B expression and the infiltration levels of 24 immune cells (Fig 6B). Moreover, we analyzed the differences of infiltration levels of immune cells between STK17B high- and low-expression groups (Fig 7A). Immune score analysis showed that STK17B expression was positively correlated with immune score, stromal score, and ESTIMATE score (Fig 7B–7D). Accordingly, these three scores were higher in patients with STK17B high-expression than low-expression (S1A–S1C Fig). STK17B expression was different in different immune subtypes (S1D Fig). These findings suggested that STK17B played an important role in immune cells infiltration of tumor microenvironment in SKCM.

### 3.7. Identification of DEGs between STK17B high- and low-expression groups

**Fig 8:**
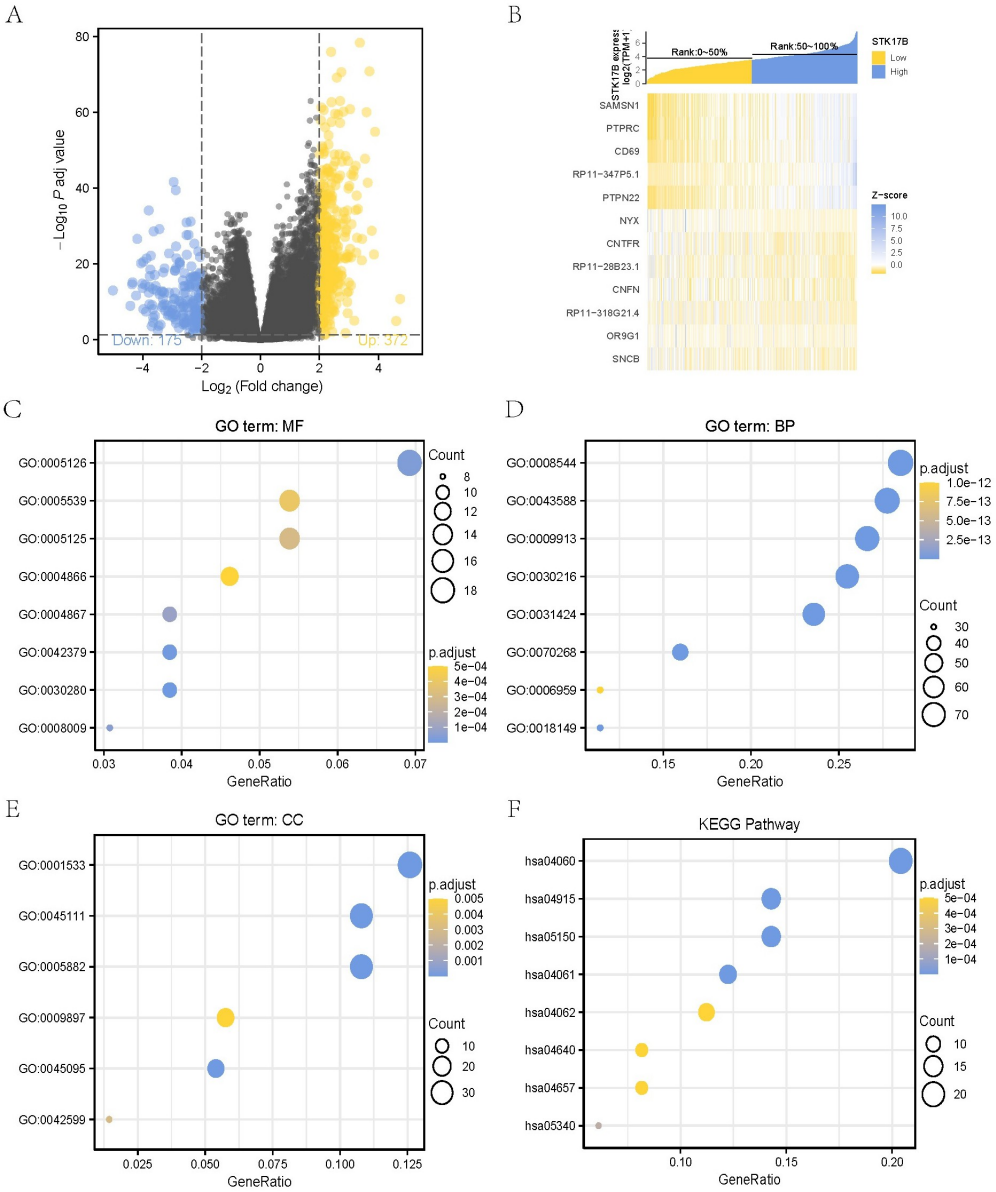
Differential expression genes between STK17B high- and low-expression groups. (**A**) A volcano plot of differential expression genes between STK17B high- and low-expression groups; (**B**) a heat map of the top 12 significant genes positively and negatively correlated with STK17B; Gene Ontology (**C-E**) and Kyoto Encyclopedia of Genes and Genomes (**F**) analyses of STK17B related differential expression genes.

To gain the insight of STK17B biological meaning in SKCM, an RNA-seq analysis was used to compare the gene expression profiles of STK17B high- and low-expression groups in TCGA database. Based on “edgeR” in R software, 547 genes expression associated with STK17B in SKCM were screened out, including 372 up-regulated genes and 175 down-regulated genes (Fig 8A). The top 12 significant genes positively and negatively correlated with STK17B were shown in the heat map (Fig 8B).

### 3.8. Functional enrichment of STK17B-related genes in SKCM

To further explore the function of the DEGs and identify key candidate pathways, GO functional analysis and KEGG pathway analysis was performed. We selected the top GO terms of the molecular function, biological process, cellular component (Fig 8C–8E). The enriched terms included cytokine receptor binding, glycosaminoglycan binding, cytokine activity, epidermis development, skin development, epidermal cell differentiation, keratinocyte differentiation, cornified envelope, intermediate filament cytoskeleton (S1 Table). In the TCGA cohort, KEGG pathways, such as cytokine-cytokine receptor interaction, estrogen signaling pathway, viral protein interaction with cytokine and cytokine receptor, which were most significantly enriched in SKCM patients with higher expression compared to lower expression (Fig 8F, S1 Table). GO and KEGG analysis revealed that STK17B might mediate the process of cellular differentiation and immunity, which consisted with the results of immune infiltration analyses.

### 3.9. Gene set enrichment analysis (GSEA) and a PPI network

**Fig 9:**
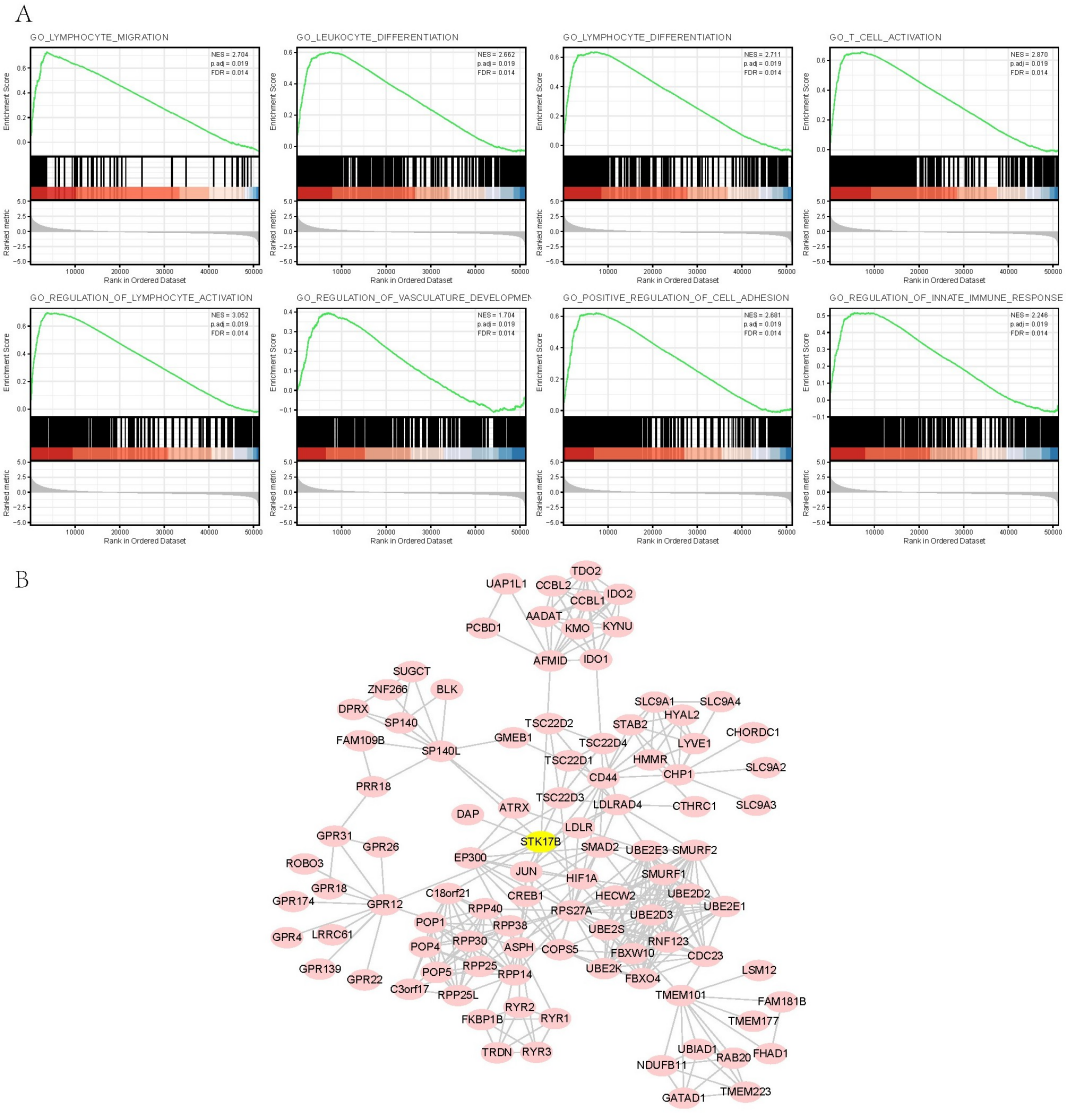
Gene set enrichment analysis of significantly enriched gene sets between STK17B high- and low-expression groups and a protein-protein interaction network. (**A**) Gene set enrichment analysis of significantly enriched gene sets between STK17B high- and low-expression groups; (**B**) a protein-protein interaction network of STK17B related differential expression genes.

GSEA was performed between STK17B high- and low-expression group. Results of GSEA revealed significant differences in MSigDB collection enrichment (c5.all.v7.0.symbols.gmt [Gene ontology]). As shown in Fig 9, the results suggested that high expression of STK17B may be highly enriched in regulation of innate immune response, leukocyte differentiation, lymphocyte migration, T cell activation, lymphocyte differentiation, regulation of lymphocyte activation, regulation of vasculature development, and positive regulation of cell adhesion, revealing that STK17B was related to immunity and cancer development. To further investigate the role of the STK17B in the development of SKCM, we constructed a PPI network by STRING to evaluate the interaction between the relevant genes (Fig 9).

## 4. Discussion

SKCM is still a refractory disease, which is also the leading cause of death from skin cancer [20]. The advanced patients’ survival has been prolonged by current clinical therapeutic methods. Nevertheless, many patients were observed subsequent resistances to the drugs and developing progressive diseases [21]. The 5-year survival of patients with metastatic melanoma is only 23%, less than a quarter of patients with localized disease [22]. It’s urgent to identify more effective diagnostic and prognostic biomarkers. STK17B, which has high expression in lymphoid tissue, has been indicated associated with hepatocellular carcinoma and some other cancers by previous studies. However, the clinical value of STK17B for SKCM is undefined. In this research, we mainly investigated the role of STK17B on prognosis, clinicopathologic features, and immune-related characteristics of SKCM.

STK17B is a death-associated protein kinase and involved in apoptosis in a variety of cell types [23, 24]. In our study, we analyzed STK17B expression data in SKCM samples obtained from TCGA database and found that the low expression level of STK17B was relevant to a poor prognosis. Radiation therapy is widely used as an adjuvant therapy for patients with advanced melanoma. It was found that radiation could increase tumor associated antibodies and the diversity of T-cell receptor [25, 26]. Our research revealed that STK17B expression was closely associated to the infiltration level of immune cells and regulated immune-related progresses. STK17B could not be a potential biomarker to predict prognosis in SKCM patients with radiation therapy because STK17B-related immune progress was changed under radiation treatment. Previous studies have demonstrated that STK17B could be an independent prognostic factor in chronic lymphocytic leukemia. The results also showed that the low STK17B expression level was significantly linked to the shorter patients’ OS [27]. On the contrast, STK17B is highly expressed in hepatocellular carcinoma (HCC) tissues, which was considered predicting a poor prognosis [6]. Different effects of STK17B expression level on prognosis might be due to the disparate pathogenesis and the correlation between STK17B and prognosis in diverse diseases need more statistical verification. Analyses of this research revealed that expression of STK17B was in obvious connection with OS and DSS for SKCM so that it could be regarded as a new prognostic biomarker of this cancer for further clinical study.

As we know, clinicopathological features presented by patients are closely related to the development and prognosis of the disease. To further explore the value of STK17B in the diagnosis and treatment of SKCM, we analyzed the relationship of STK17B with clinicopathological features. The results indicated that STK17B was correlated with T stage, Breslow depth and radiation therapy in SKCM patients. It has been identified that high expression level of STK17B in HCC was strikingly related to poor clinicopathological feature, including tumor size, TNM stage, and venous invasion [6]. Similarly, the significant correlations can also be found between the abnormal expression of STK17B and clinicopathologic variables in non-Hodgkin’s lymphoma [28]. Above results suggested that STK17B was an important factor influencing clinicopathological characteristics and could be a possible tumor biomarker and drug target. To a certain extent, the nomogram could predict the prognosis of SKCM patients.

Tumor-infiltrating immune cells constitute a part of the tumor microenvironment and play an important role in regulating tumor development and progression. Previous researches have revealed that STK17B highly expresses in B and T cells and negatively regulates activated T cells [29, 30]. In this research, we noticed that STK17B expression level was positively correlated with infiltration levels of Th cells, T cells, Th1 cells, Tcm, and Th2 cells by estimating the association of STK17B expression and immune cells. Previous study revealed that IL-9-producing CD4+ T cells, a type of T helper cells, had potent abilities in eradicating melanoma [31]. Results of our study showed that STK17B expression was significantly associated to infiltration level of T helper cells. According to prognosis analysis results of our research, higher STK17B expression indicated longer overall survival and disease-specific survival. The underlying mechanism might be high STK17B expression leading to high infiltration level of immune cells which contribute to resist tumor cells. Therefore, high STK17B expression suggest good prognosis. It has been found that STK17B affected autoimmune diseases via regulating T cell survival [32]. Besides, the breast cancer cell line with depletion of STK17B retarded tumorigenesis and inhibited tumor growth in a xenograft model [5]. However, Benjamin A. Edwards, et al. deemed that STK17B was not a necessary tumor-inhibiting factor or an oncogene. Take into consideration that STK17B expresses differently in humans and mice, the mechanism and consequences should also be distinguishing. It is also possible that tissue specificity causes STK17B to have different mechanisms in SKCM. STK17B mediates SKCM related immune mechanisms in other undiscovered ways. After all, it’s undoubted that STK17B is significantly linked to immunological process.

Staphylococcus aureus (S. aureus) is a common pathogenic factor of bloodstream infection, which has a strong impact on mortality of cancer patients [33]. However, it has also been clarified that S. aureus infection is associated with low melanoma risk. The mechanism could be that immune response caused by S. aureus infection also identifies tumor cells and kill them during defensing S. aureus, suggesting that S. aureus infection is closely linked to morbidity and mortality in melanoma patients [34]. The enrichment results of our study revealed that differential expression of STK17B in melanoma patients was related to S. aureus infection. Our study also demonstrated the correlation between STK17B expression and immune-related progress in melanoma patients. Different expression level of STK17B might regulate the sensibility to S. aureus and influence prognosis of SKCM patients.

All above analyses of this research were based on the information from TCGA and GTEx databases, which emphasized on theoretical research. It is not known whether our results consist with experimental verification. In vitro and in vivo experiments are required to clarify the influence of STK17B on the development of SKCM. The underlying mechanisms need to be ascertained to prove the value of STK17B in settling clinical practical problems and rationalize the different results of other studies. On this basis, the therapeutic effect of STK17B on SKCM can be further studied.

In summary, we studied the relationship between STK17B and SKCM for the first time. We discovered that lower STK17B expression was connected with shorter OS and DSS in SKCM patients. Moreover, this gene was likewise related to clinicopathologic features and immune-related characteristics of SKCM. These results connote STK17B may be a molecular targets and lead to new discovery of treatments for SKCM.

## Supporting information

S1 Fig Immune score of STK17B expression and immune subtype analysis. Stromal score (**A**), immune score (**B**), and ESTIMATE score (**C**) of STK17B high- and low-expression groups; (**D**) STK17B expression level in different immune subtypes.
(TIF)

S1 Table Enrichment analyses of STK17B-related genes in skin cutaneous melanoma. (DOCX)
