# Supplementary figures and images for: Prognostic and immune-related value of STK17B in skin cutaneous melanoma
Source: PLoS One. 2022 Feb 16;17(2):e0263311. doi: 10.1371/journal.pone.0263311 (PMC8849620; doi:10.1371/journal.pone.0263311)

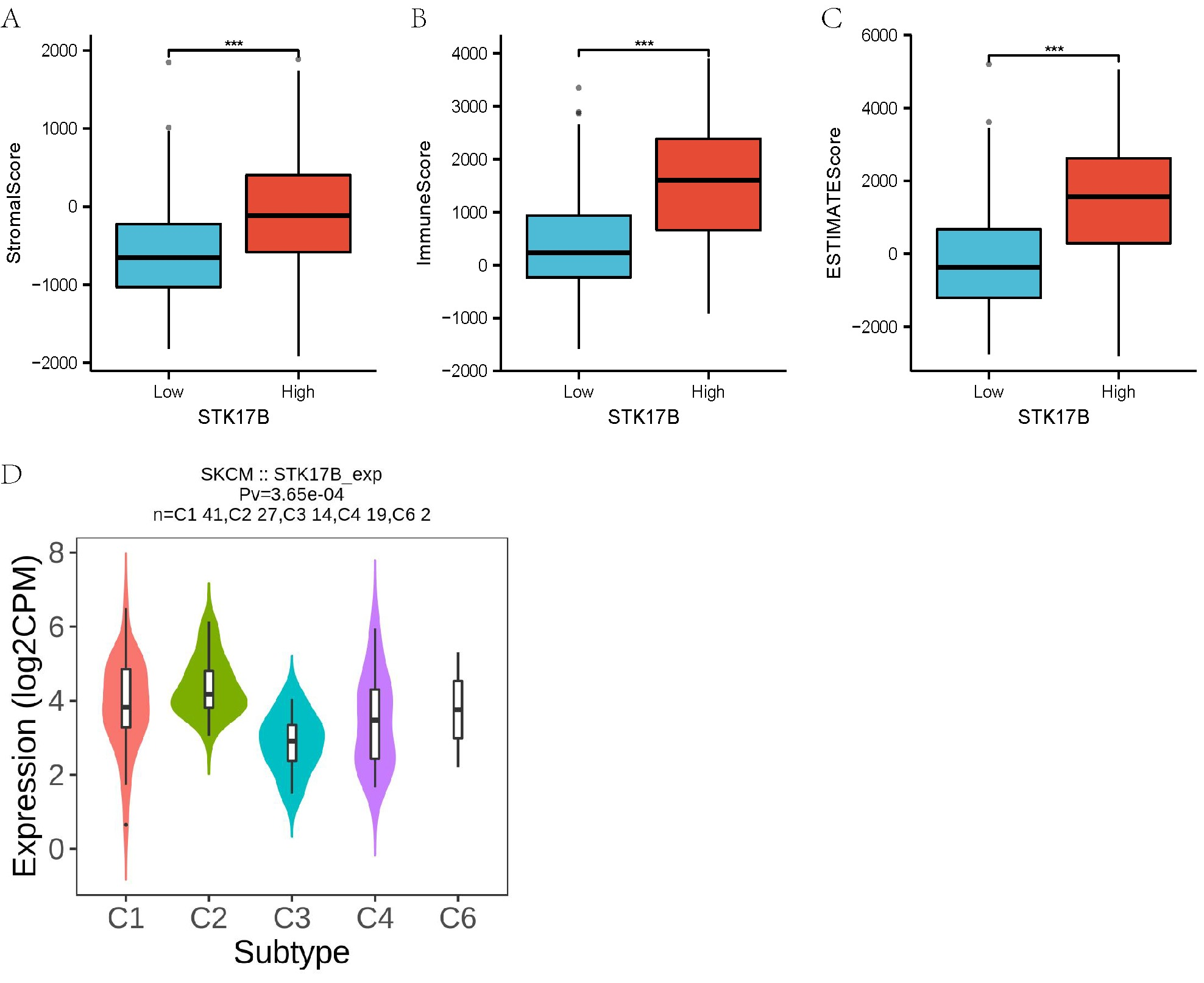

Supplement: S1 Fig — Stromal score (A), immune score (B), and ESTIMATE score (C) of STK17B high- and low-expression groups; (D) STK17B expression level in different immune subtypes. (TIF) [file pone.0263311.s001.tif]
