# Supplementary material for: Prognostic and immune-related value of STK17B in skin cutaneous melanoma
Source: PLoS One. 2022 Feb 16;17(2):e0263311. doi: 10.1371/journal.pone.0263311 (PMC8849620; doi:10.1371/journal.pone.0263311)
Supplement: S1 Table — (DOCX) [file pone.0263311.s002.docx]

| ID | Description | pvalue | p.adjust | geneID | Count |
| --- | --- | --- | --- | --- | --- |
| MF |  |  |  |  |  |
| GO:0030280 | structural constituent of epidermis | 2.92E-15 | 1.05E-12 | KRT1/PKP1/PI3/FLG/SPRR2E/SPRR1A/FLG2/LOR/KRT2/KRT82 | 10 |
| GO:0042379 | chemokine receptor binding | 4.04E-08 | 7.2854E-06 | CXCL9/CCL21/CXCL10/CXCL13/CXCL11/S100A14/CCL20/CXCL5/C10orf99/DEFB4A | 10 |
| GO:0005126 | cytokine receptor binding | 2.51E-07 | 0 | CXCL9/CCL21/CXCL10/CXCL13/CXCL11/S100A14/CNTFR/BMP2/CCL20/CXCL5/C10orf99/STAP1/TNFSF11/IFNG/RHEX/DEFB4A/IL21/IL2 | 18 |
| GO:0008009 | chemokine activity | 5.22E-07 | 0 | CXCL9/CCL21/CXCL10/CXCL13/CXCL11/CCL20/CXCL5/C10orf99 | 8 |
| GO:0004867 | serine-type endopeptidase inhibitor activity | 1.2062E-06 | 0.0001 | PI3/SLPI/A2ML1/SERPINB3/SERPINB4/SERPINB13/WFDC12/WFDC5/SPINK7/SERPINB12 | 10 |
| GO:0005125 | cytokine activity | 4.8863E-06 | 0.0003 | CXCL9/CCL21/CXCL10/CXCL13/CXCL11/BMP2/CCL20/CXCL5/C10orf99/TNFSF11/IFNG/SLURP1/IL21/IL2 | 14 |
| GO:0005539 | glycosaminoglycan binding | 7.7486E-06 | 0.0004 | JCHAIN/PTPRC/CXCL10/CXCL13/ABI3BP/PLA2G2D/CXCL11/HAPLN1/FGFBP1/RNASE7/PGLYRP4/PGLYRP3/FGF10/ELSPBP1 | 14 |
| GO:0004866 | endopeptidase inhibitor activity | 0 | 0.0005 | PI3/SLPI/A2ML1/SERPINB3/SERPINB4/SERPINB13/WFDC12/WFDC5/CARD18/SPINK7/SERPINB12/SMR3B | 12 |
| BP |  |  |  |  |  |
| GO:0031424 | keratinization | 1.89E-63 | 4.83E-60 | KRT14/KRT6A/KRT16/KRT5/KRT17/KRT6B/KRT1/KRT6C/SFN/PKP1/SPRR1B/PI3/FLG/CASP14/IVL/EVPL/SPRR2E/CNFN/SPRR2G/SPRR1A/SPRR2A/KLK5/TGM1/LOR/KRT2/LCE3D/KRT75/KLK13/LCE1C/LCE3E/SPRR2B/CDSN/LCE1A/LCE2B/LCE1B/LCE2C/LCE1F/KRT9/SPRR4/LCE6A/LCE2D/LCE1D/LCE3A/KRT34/LCE1E/KRT71/KRT85/LCE5A/KRT25/LCE3C/KRT35/KRTAP11-1/KRT74/LCE4A/KRT83/KRT82/KRT28/KRT33B/KRTAP19-5/KRT33A/KRT37/KRTAP3-1 | 62 |
| GO:0030216 | keratinocyte differentiation | 2.58E-61 | 3.30E-58 | KRT14/KRT6A/KRT16/KRT5/KRT17/KRT6B/KRT1/KRT6C/SFN/PKP1/SPRR1B/PI3/S100A7/FLG/CASP14/IVL/EVPL/SPRR2E/CNFN/SPRR2G/SPRR1A/SPRR2A/KLK5/TGM1/LOR/KRT2/LCE3D/KRT75/KLK13/SERPINB13/LCE1C/LCE3E/SPRR2B/CDSN/LCE1A/LCE2B/LCE1B/LCE2C/LCE1F/FOXN1/C1orf68/KRT9/SPRR4/ACER1/LCE6A/LCE2D/LCE1D/LCE3A/KRT34/LCE1E/KRT71/KRT85/LCE5A/KRT25/LCE3C/KRT35/KRTAP11-1/KRT74/LCE4A/KRT83/KRT82/KRT28/KRT33B/KRTAP19-5/KRT33A/KRT37/KRTAP3-1 | 67 |
| GO:0009913 | epidermal cell differentiation | 2.17E-60 | 1.85E-57 | KRT14/KRT6A/KRT16/KRT5/KRT17/KRT6B/KRT1/KRT6C/SFN/PKP1/SPRR1B/PI3/S100A7/FLG/CASP14/IVL/EVPL/SPRR2E/CNFN/SPRR2G/SPRR1A/SPRR2A/KLK5/TGM1/LOR/SULT2B1/KRT2/LCE3D/KRT75/KLK13/OVOL1/SERPINB13/LCE1C/LCE3E/SPRR2B/CDSN/LCE1A/LCE2B/LCE1B/LCE2C/LCE1F/FOXN1/C1orf68/KRT9/SPRR4/ACER1/LCE6A/LCE2D/LCE1D/LCE3A/KRT34/LCE1E/KRT71/KRT85/LCE5A/KRT25/LCE3C/KRT35/KRTAP11-1/KRT74/LCE4A/KRT83/KRT82/KRT28/KRT33B/KRTAP19-5/CLRN1/KRT33A/KRT37/KRTAP3-1 | 70 |
| GO:0043588 | skin development | 2.88E-59 | 1.84E-56 | KRT14/KRT6A/KRT16/KRT5/KRT17/KRT6B/KRT1/KRT6C/SFN/PKP1/SPRR1B/PI3/S100A7/FLG/CASP14/IVL/EVPL/SPRR2E/CNFN/ASPRV1/SPRR2G/SPRR1A/SPRR2A/KLK5/TGM1/FLG2/LOR/KRT2/LCE3D/KRT75/KLK13/OVOL1/ALOX12B/SERPINB13/LCE1C/LCE3E/SPRR2B/ALOXE3/CDSN/LCE1A/LCE2B/LCE1B/LCE2C/LCE1F/FOXN1/C1orf68/KRT9/SPRR4/ACER1/FGF10/LCE6A/LCE2D/LCE1D/LCE3A/KRT34/LCE1E/KRT71/KRT85/LCE5A/KRT25/LCE3C/KRT35/KRTAP11-1/KRT74/LCE4A/KRT83/KRT82/KRT28/KRT33B/KRTAP19-5/KRT33A/KRT37/KRTAP3-1 | 73 |
| GO:0008544 | epidermis development | 1.86E-58 | 9.53E-56 | KRT14/KRT6A/KRT16/KRT5/KRT17/KRT6B/KRT1/KRT6C/SFN/PKP1/SPRR1B/PI3/S100A7/FLG/CALML5/KRTDAP/CASP14/IVL/EVPL/SPRR2E/CNFN/SPRR2G/SPRR1A/SPRR2A/KLK5/TGM1/FLG2/KLK7/LOR/SULT2B1/KRT2/LCE3D/KRT75/KLK13/OVOL1/SERPINB13/LCE1C/LCE3E/SPRR2B/CDSN/LCE1A/LCE2B/LCE1B/LCE2C/LCE1F/FOXN1/C1orf68/KRT9/SPRR4/ACER1/FGF10/LCE6A/LCE2D/LCE1D/LCE3A/KRT34/LCE1E/KRT71/KRT85/LCE5A/KRT25/LCE3C/KRT35/KRTAP11-1/KRT74/LCE4A/KRT83/KRT82/KRT28/KRT33B/KRTAP19-5/CLRN1/KRT33A/KRT37/KRTAP3-1 | 75 |
| GO:0070268 | cornification | 3.03E-49 | 1.29E-46 | KRT14/KRT6A/KRT16/KRT5/KRT17/KRT6B/KRT1/KRT6C/PKP1/SPRR1B/PI3/FLG/CASP14/IVL/EVPL/SPRR2E/SPRR2G/SPRR1A/SPRR2A/KLK5/TGM1/LOR/KRT2/LCE3D/KRT75/KLK13/SPRR2B/CDSN/LCE1A/KRT9/KRT34/KRT71/KRT85/KRT25/KRT35/KRT74/KRT83/KRT82/KRT28/KRT33B/KRT33A/KRT37 | 42 |
| GO:0018149 | peptide cross-linking | 4.38E-40 | 1.60E-37 | KRT1/SPRR1B/PI3/FLG/IVL/EVPL/SPRR2E/SPRR1A/SPRR2A/TGM1/LOR/KRT2/LCE3D/LCE1C/LCE3E/SPRR2B/LCE1A/LCE2B/LCE1B/LCE2C/LCE1F/C1orf68/SPRR4/LCE2D/LCE1D/LCE3A/LCE1E/LCE5A/LCE3C/LCE4A | 30 |
| GO:0006959 | humoral immune response | 3.70E-15 | 1.18E-12 | KRT6A/CXCL9/JCHAIN/KRT1/IGLL5/PTPRC/CXCL10/CXCL13/MS4A1/PI3/S100A7/CR2/CXCL11/PAX5/SLPI/CD19/KLK5/FCER2/SH2D1A/CR1/KLK7/CXCL5/IFNG/HTN3/RNASE7/PGLYRP4/PGLYRP3/DEFB4A/IGLL1/CCR6 | 30 |
| CC |  |  |  |  |  |
| GO:0001533 | cornified envelope | 3.85E-48 | 7.20E-46 | KRT1/PKP1/SPRR1B/PI3/FLG/IVL/EVPL/SPRR2E/CNFN/SPRR2G/SPRR1A/SPRR2A/TGM1/FLG2/LOR/KRT2/LCE3D/LCE1C/LCE3E/SPRR2B/CDSN/LCE1A/LCE2B/LCE1B/LCE2C/LCE1F/C1orf68/SPRR4/LCE2D/LCE1D/LCE3A/LCE1E/LCE5A/LCE3C/LCE4A | 35 |
| GO:0005882 | intermediate filament | 2.29E-21 | 2.14E-19 | KRT14/KRT6A/KRT16/KRT5/KRT17/KRT6B/KRT1/KRT6C/PKP1/FLG/CASP14/EVPL/KRT2/KRT75/KRT9/KRT34/KRT71/KRT85/KRT25/KRT35/KRTAP11-1/KRT74/KRT83/KRT82/KRT28/KRT33B/KRTAP19-5/KRT33A/KRT37/KRTAP3-1 | 30 |
| GO:0045111 | intermediate filament cytoskeleton | 2.41E-19 | 1.50E-17 | KRT14/KRT6A/KRT16/KRT5/KRT17/KRT6B/KRT1/KRT6C/PKP1/FLG/CASP14/EVPL/KRT2/KRT75/KRT9/KRT34/KRT71/KRT85/KRT25/KRT35/KRTAP11-1/KRT74/KRT83/KRT82/KRT28/KRT33B/KRTAP19-5/KRT33A/KRT37/KRTAP3-1 | 30 |
| GO:0045095 | keratin filament | 4.77E-12 | 2.23E-10 | KRT14/KRT6A/KRT5/KRT6B/KRT1/KRT6C/CASP14/KRT2/KRT75/KRT71/KRT85/KRT74/KRT83/KRT82/KRTAP3-1 | 15 |
| GO:0042599 | lamellar body | 0.0001 | 0.003 | KRTDAP/KLK5/KLK7/SFTPB | 4 |
| GO:0009897 | external side of plasma membrane | 0.0002 | 0.0049 | CXCL9/IGLL5/PTPRC/CXCL10/MS4A1/IL7R/CD3G/CD19/CNTFR/FCER2/CD69/LY6G6C/CCR4/CXCR5/IGLL1/CCR6 | 16 |
| KEGG |  |  |  |  |  |
| hsa05150 | Staphylococcus aureus infection | 6.15E-12 | 1.03E-09 | KRT14/KRT16/KRT17/KRT9/DEFB4A/DEFB4B/KRT34/LOC100653049/KRT25/KRT35/KRT28/KRT33B/KRT33A/KRT37 | 14 |
| hsa04060 | Cytokine-cytokine receptor interaction | 2.31E-10 | 1.93E-08 | CXCL9/CCL21/CXCL10/CXCL13/IL7R/CXCL11/CNTFR/BMP2/CCL20/TNFRSF17/CXCL5/CCR4/TNFSF11/IFNG/TNFRSF13B/IL18RAP/CXCR5/CCR6/IL21/IL2 | 20 |
| hsa04915 | Estrogen signaling pathway | 8.95E-10 | 4.98E-08 | KRT14/KRT16/KRT17/CALML5/CALML3/KRT9/KRT34/LOC100653049/KRT25/KRT35/KRT28/KRT33B/KRT33A/KRT37 | 14 |
| hsa04061 | Viral protein interaction with cytokine and cytokine receptor | 2.24E-09 | 9.35E-08 | CXCL9/CCL21/CXCL10/CXCL13/CXCL11/CCL20/CXCL5/CCR4/IL18RAP/CXCR5/CCR6/IL2 | 12 |
| hsa05340 | Primary immunodeficiency | 5.5246E-06 | 0.0002 | PTPRC/IL7R/CD19/TNFRSF13B/AICDA/IGLL1 | 6 |
| hsa04657 | IL-17 signaling pathway | 0 | 0.0005 | CXCL10/S100A7/CCL20/S100A7A/CXCL5/IFNG/DEFB4A/DEFB4B | 8 |
| hsa04062 | Chemokine signaling pathway | 0 | 0.0005 | CXCL9/CCL21/CXCL10/CXCL13/CXCL11/RASGRP2/CCL20/CXCL5/CCR4/CXCR5/CCR6 | 11 |
| hsa04640 | He+A1:B35matopoietic cell lineage | 0 | 0.0005 | MS4A1/IL7R/CR2/CD3G/CD19/FCER2/CR1/DNTT | 8 |
